# Predicting Renal Recovery After Dialysis-Requiring Acute Kidney Injury

**DOI:** 10.1016/j.ekir.2019.01.015

**Published:** 2019-01-28

**Authors:** Benjamin J. Lee, Chi-yuan Hsu, Rishi Parikh, Charles E. McCulloch, Thida C. Tan, Kathleen D. Liu, Raymond K. Hsu, Leonid Pravoverov, Sijie Zheng, Alan S. Go

**Affiliations:** 1 Division of Nephrology, Department of Medicine, University of California, San Francisco, San Francisco, California, USA; 2 Houston Kidney Consultants, Houston, Texas, USA; 3 Houston Methodist Institute for Academic Medicine, Houston, Texas, USA; 4 Division of Research, Kaiser Permanente Northern California, Oakland, California, USA; 5 Department of Epidemiology and Biostatistics, University of California, San Francisco, San Francisco, California, USA; 6 Division of Critical Care, Department of Anesthesia, University of California, San Francisco, San Francisco, California, USA; 7 Department of Nephrology, Kaiser Permanente Oakland Medical Center, Oakland, California, USA

**Keywords:** dialysis-requiring acute kidney injury, prediction model, renal recovery

## Abstract

**Introduction:**

After dialysis-requiring acute kidney injury (AKI-D), recovery of sufficient kidney function to discontinue dialysis is an important clinical and patient-oriented outcome. Predicting the probability of recovery in individual patients is a common dilemma.

**Methods:**

This cohort study examined all adult members of Kaiser Permanente Northern California who experienced AKI-D between January 2009 and September 2015 and had predicted inpatient mortality of <20%. Candidate predictors included demographic characteristics, comorbidities, laboratory values, and medication use. We used logistic regression and classification and regression tree (CART) approaches to develop and cross-validate prediction models for recovery.

**Results:**

Among 2214 patients with AKI-D, mean age was 67.1 years, 40.8% were women, and 54.0% were white; 40.9% of patients recovered. Patients who recovered were younger, had higher baseline estimated glomerular filtration rates (eGFR) and preadmission hemoglobin levels, and were less likely to have prior heart failure or chronic liver disease. Stepwise logistic regression applied to bootstrapped samples identified baseline eGFR, preadmission hemoglobin level, chronic liver disease, and age as the predictors most commonly associated with coming off dialysis within 90 days. Our final logistic regression model including these predictors had a correlation coefficient between observed and predicted probabilities of 0.97, with a c-index of 0.64. An alternate CART approach did not outperform the logistic regression model (c-index 0.61).

**Conclusion:**

We developed and cross-validated a parsimonious prediction model for recovery after AKI-D with excellent calibration using routinely available clinical data. However, the model’s modest discrimination limits its clinical utility. Further research is needed to develop better prediction tools.

See Commentary on Page 520

AKI-D is a serious acute medical condition that affects 3% to 13% of critically ill patients.^1^, ^2^, ^3^ Although in-hospital mortality among patients with AKI-D has declined,^4^, ^5^, ^6^, ^7^, ^8^ a sizeable fraction of survivors remain dialysis-dependent at the time of hospital discharge and beyond.^2^, ^5^, ^9^, ^10^, ^11^ Renal recovery after AKI-D, defined as return of sufficient kidney function to discontinue dialysis, is an important clinical and patient-oriented outcome. Although most patients with normal baseline kidney function eventually recover if they survive the AKI-D hospitalization,^12^ many patients with AKI-D experience acute kidney injury (AKI) superimposed on chronic kidney disease (CKD) and do not recover.^13^, ^14^, ^15^

Prediction of recovery after the onset of AKI-D is a common dilemma confronted by patients, their families, and physicians across multiple specialties, from nephrologists to intensivists, hospitalists, and primary care physicians. Baseline eGFR, proteinuria, age, diabetes mellitus, and comorbidity burden have been shown to influence the probability of recovery.^9^, ^10^, ^13^, ^14^, ^15^, ^16^, ^17^ The only published prediction model was constructed by Srisawat *et al.*,^18^ who found that Charlson comorbidity index and APACHE II score were predictors. However, their study was small (*n* = 76) and included only highly selected participants enrolled in a clinical trial that excluded patients with preexisting stage 4 or 5 CKD, so generalizability was limited for multiple reasons.^19^, ^20^ Overall, data on the natural history of AKI-D are variable, and it is difficult to know whether an individual patient with AKI-D will recover.^9^, ^21^

The ability to predict recovery more accurately could potentially guide counseling and decision-making in both the inpatient and outpatient settings. Many hospitalized patients with AKI ask about their chances of recovery even before initiating acute dialysis, and some may decline starting dialysis altogether if they understand that the chances of recovery are very low and they will likely be on dialysis for the rest of their lives. Accurate prediction of recovery would inform dialysis access decisions for patients with AKI-D: both the choice of temporary versus tunneled catheters in the short-term and the timing of fistula or graft placement in the medium-term. Improved prognostic abilities would also influence the timing of outpatient dialysis chair placement (i.e., establishing a time and location for outpatient dialysis), which could potentially affect hospital length of stay. In the outpatient setting, when patients consider procedures that may prolong AKI-D (e.g., iodinated contrast administration), the ability to predict recovery would help patients and their providers appropriately weigh risks and benefits. From a research perspective, improved prognostic abilities would allow for targeted enrollment of patients with AKI-D who have a reasonable chance of recovery into trials testing potential treatments.

There are currently no validated AKI-D recovery prediction models, and expert panels have identified this knowledge gap as a key barrier to improving outcomes in this vulnerable population.^9^, ^22^ Using a diverse, community-based cohort, our objective was to develop a prediction model for recovery after AKI-D that would be applicable to routine clinical practice.

## Methods

### Source Population

The source population was based within Kaiser Permanente Northern California (KPNC), a large, integrated health care delivery system that provides comprehensive care for >4.4 million members. These patients were treated in 21 Kaiser Permanente–owned hospitals (Supplementary Appendix S1). The KPNC membership is highly representative of the surrounding local and statewide populations.^23^ Nearly all aspects of care are captured through KPNC’s electronic medical record system, which is integrated across inpatient, emergency department, and outpatient care settings.

This study was approved by the institutional review boards at KPNC and the University of California, San Francisco, with waiver of informed consent obtained because of the nature of the study.

### Study Sample

We conducted a retrospective cohort study of all adult (age ≥18 years) KPNC members who developed AKI-D between January 1, 2009, and September 30, 2015, and who had ≥12 consecutive months of health plan membership and pharmacy benefits before the index hospitalization to ensure adequate capture of relevant comorbidities, laboratory tests, and prescription medication use. For this analysis, we classified patients as having AKI-D if they underwent renal replacement therapy (RRT; acute intermittent hemodialysis and/or continuous RRT) during hospitalization in the absence of any preadmission chronic RRT *and* had peak inpatient serum creatinine concentration ≥50% of preadmission baseline (defined as the most recent non–emergency department outpatient measurement between 7 and 365 days before admission). Chronic RRT before admission was ascertained through a comprehensive KPNC End-Stage Renal Disease (ESRD) Treatment Registry that tracks initiation and cessation of RRT treatments and date(s) of renal transplantation.^13^, ^15^, ^24^, ^25^ We excluded patients who had baseline eGFR values <15 ml/min per 1.73 m^2^ (because it is difficult in this eGFR range to distinguish true AKI-D from progression of severe CKD) or predicted probability of inpatient mortality ≥20% using a KPNC-validated risk score^26^ (because the issue of renal recovery is clinically relevant only among those patients with AKI-D who are likely to survive the acute hospitalization and also to reduce analytic issues introduced when death can be interpreted as a state of “nonrecovery” after AKI-D).

We also conducted 2 sensitivity analyses: one that did not exclude patients with predicted probability of inpatient mortality ≥20% and one that used serum creatinine instead of eGFR.

### Renal Recovery After AKI-D

The primary outcome was recovery of native kidney function after AKI-D, defined as RRT independence within 90 days after RRT initiation and survival for ≥4 weeks after RRT discontinuation. Patients who stopped RRT within 4 weeks of the 90-day cutoff were observed past 90 days to confirm that they remained alive for the minimum 4-week period. We used status at 90 days because patients are conventionally considered to have ESRD if they remain dialysis-dependent for ≥90 days.^9^ We required that patients be alive and off dialysis for ≥4 weeks to reduce potential misclassification of people who discontinued dialysis due to withdrawal of care. Recovery could occur during the initial AKI-D hospitalization or in the outpatient setting after hospital discharge. We anchored our analysis based on the date of RRT initiation (rather than hospital discharge or some other date) to link it more closely to the natural history of the AKI episode rather than other extraneous factors that may influence length of hospitalization.

### Covariates

Demographic characteristics (e.g., age, gender, self-reported race and ethnicity) were obtained from health plan databases.^27^, ^28^, ^29^ Relevant comorbidities were defined by diagnostic or procedural *International Classification of Diseases, Ninth Revision* codes and supplemented with laboratory test results, outpatient vital signs, and prescribed medications using electronic health record–based data that were cleaned and linked at the individual-patient level into the Kaiser Permanente Virtual Data Warehouse as previously described and validated.^25^, ^30^, ^31^, ^32^, ^33^, ^34^, ^35^, ^36^, ^37^, ^38^ Patient vital status was determined using comprehensive information from health plan administrative and clinical databases, member proxy reporting, Social Security Administration vital status files, and California state death certificate information.^39^, ^40^ Demographic characteristics and inpatient laboratory values were measured on the date of RRT initiation for AKI-D, and baseline outpatient laboratory values and vital signs were measured 7 to 365 days before admission. For variables that had missing data, a category for missingness was created for each of those variables. Variables with >20% of values missing were not included in the modeling process.

### Statistical Approach

Analyses were conducted using SAS, version 9.3 (SAS Inc., Cary, NC) and Salford Predictive Modeler, version 8.2 (Salford Systems, San Diego, CA). Baseline characteristics were compared across recovery groups using analysis of variance for continuous variables and χ^2^ tests for categorical variables.

We initially conducted a multivariable logistic regression analysis for prediction of recovery after AKI-D, with the following candidate predictors: age, gender, self-reported race and ethnicity, smoking status, preadmission medication use, preexisting comorbidities (heart failure, coronary heart disease, prior ischemic stroke, peripheral artery disease, atrial fibrillation, mitral or aortic valvular disease, venous thromboembolism, hypertension, diabetes mellitus, dyslipidemia, prior hospitalized gastrointestinal bleed, thyroid disease, chronic liver disease, chronic lung disease, dementia, depression), and inpatient mortality risk score.^26^ Additional candidate predictors included the following preadmission variables: body mass index, systolic blood pressure, preadmission high-density and low-density lipoprotein levels, eGFR (using the Chronic Kidney Disease Epidemiology Collaboration creatinine equation^41^), dipstick proteinuria level, hemoglobin level, and platelet count. Body mass index, preadmission systolic blood pressure, and all laboratory-based variables were treated as ordinal categorical variables (partitions between categories shown in Tables 1 and 2; similar results were obtained when these covariates were treated as continuous variables). To identify important predictors, we first generated 1000 random samples of the analytic cohort through bootstrap resampling with replacement and then conducted automated stepwise logistic regression on each sample. Predictors that were selected by stepwise regression in ≥75% of the bootstrapped samples were included in the final model. We subsequently used 10-fold cross-validation to generate predicted probabilities of recovery for each patient, which were used to calculate a c-index and generate calibration statistics. Finally, model parameter estimates and odds ratios for the final set of predictors were generated through a logistic regression model using the full analytic cohort.

**Table 1:** Selected baseline characteristics of adults with dialysis-requiring acute kidney injury, stratified by renal recovery status

Variable^a^	Overall	Not recovered	Recovered	*P*
	(*N* = 2214)	(*n* = 1309)	(*n* = 905)	
Age, yr	67.1 (13.1)	67.9 (13.1)	66.0 (13.1)	**<0.001**
Women, *n* (%)	904 (40.8)	546 (41.7)	358 (39.6)	0.31
Self-reported race, *n* (%)				0.82
White	1195 (54.0)	696 (53.2)	499 (55.1)	
Black/African American	281 (12.7)	167 (12.8)	114 (12.6)	
Asian/Pacific Islander	268 (12.1)	162 (12.4)	106 (11.7)	
Other/Unknown	470 (21.2)	284 (21.7)	186 (20.6)	
Hispanic ethnicity, *n* (%)	408 (18.4)	254 (19.4)	154 (17.0)	0.15
Medical history, *n* (%)				
Acute myocardial infarction	127 (5.7)	83 (6.3)	44 (4.9)	0.14
Coronary artery bypass graft surgery	35 (1.6)	19 (1.5)	16 (1.8)	0.56
Stroke or transient ischemic attack	90 (4.1)	55 (4.2)	35 (3.9)	0.70
Heart failure	750 (33.9)	495 (37.8)	255 (28.2)	**<0.001**
Diabetes mellitus	1269 (57.3)	770 (58.8)	499 (55.1)	0.08
Hypertension	1862 (84.1)	1108 (84.6)	754 (83.3)	0.40
Chronic liver disease	181 (8.2)	131 (10.0)	50 (5.5)	**<0.001**
Body mass index, kg/m^2^	31.3 (8.6)	30.8 (8.5)	32.1 (8.7)	**<0.001**
Predicted probability of inpatient mortality^b^	0.1 (0.0)	0.1 (0.0)	0.1 (0.0)	0.90
Preadmission medication use, *n* (%)				
ACE inhibitor	806 (36.4)	455 (34.8)	351 (38.8)	0.05
Angiotensin II receptor blocker	384 (17.3)	220 (16.8)	164 (18.1)	0.42
Diuretic	1403 (63.4)	866 (66.2)	537 (59.3)	**0.001**
Any antihypertensive agent	1944 (87.8)	1155 (88.2)	789 (87.2)	0.46
Nonsteroidal anti-inflammatory drug	186 (8.4)	92 (7.0)	94 (10.4)	**0.005**
Diabetic therapy	823 (37.2)	503 (38.4)	320 (35.4)	0.14
Laboratory values				
Preadmission eGFR, ml/min per 1.73 m^2^				
60–150	777 (35.1)	389 (29.7)	388 (42.9)	**<0.001**
45–59	367 (16.6)	190 (14.5)	177 (19.6)	
30–44	461 (20.8)	277 (21.2)	184 (20.3)	
15–29	609 (27.5)	453 (34.6)	156 (17.2)	
Preadmission creatinine, mg/dl	1.6 (0.8)	1.7 (0.8)	1.4 (0.7)	**<0.001**
Median (25th–75th percentile)	1.4 (1.0–2.1)	1.5 (1.0–2.3)	1.2 (1.0–1.8)	**<0.001**
Preadmission dipstick proteinuria, *n* (%)				
Negative/Trace	289 (13.1)	164 (12.5)	125 (13.8)	**<0.001**
1+	278 (12.6)	170 (13.0)	108 (11.9)	
≥2+	457 (20.6)	307 (23.5)	150 (16.6)	
Unknown	1190 (53.7)	668 (51.0)	522 (57.7)	
Preadmission hemoglobin, g/dl	11.7 (2.0)	11.5 (1.9)	12.1 (2.1)	**<0.001**
Preadmission serum albumin, g/dl	3.6 (0.7)	3.5 (0.7)	3.7 (0.7)	**<0.001**
Preadmission platelet count, × 10^3^/μl				**<0.001**
>400	108 (4.9)	68 (5.2)	40 (4.4)	
150–400	1421 (64.2)	823 (62.9)	598 (66.1)	
<150	399 (18.0)	270 (20.6)	129 (14.3)	
Peak inpatient serum creatinine, mg/dl	5.7 (2.8)	5.6 (2.7)	5.9 (2.9)	**0.02**

ACE, angiotensin-converting enzyme; eGFR, estimated glomerular filtration rate.

a Mean (SD) unless otherwise indicated.

b Probability range 0 to 1.

**Table 2:** Multivariable predictors of renal recovery after dialysis-requiring acute kidney injury

Variable	Adjusted odds ratio (95% CI)
Age, yr	
18–40	Ref
41–60	1.40 (0.87–2.26)
61–75	1.09 (0.68–1.73)
>75	0.85 (0.53–1.38)
Chronic liver disease	0.46 (0.32–0.65)
Preadmission CKD-EPI eGFR category, ml/min per 1.73 m^2^	
60–150	Ref
45–59	1.05 (0.81–1.36)
30–44	0.77 (0.60–0.99)
15–29	0.41 (0.32–0.53)
Preadmission hemoglobin, g/dl	
≥14	Ref
13.0–13.9	0.76 (0.53–1.08)
12.0–12.9	1.14 (0.82–1.58)
11.0–11.9	0.61 (0.44–0.85)
10.0–10.9	0.81 (0.57–1.14)
9.0–9.9	0.61 (0.42–0.89)
<9.0	0.62 (0.40–0.97)

CI, confidence interval; CKD-EPI, Chronic Kidney Disease Epidemiology Collaboration; eGFR, estimated glomerular filtration rate.

We also planned *a priori* to perform a CART analysis for recovery because it was not known whether this method would yield more clinically useful results than the logistic regression approach.^42^ Candidate predictors were the same as those used in the logistic regression analysis. CART treated all laboratory values as continuous variables and optimally selected cut-points to minimize information loss. No limits were set on minimum node or terminal size. Trees were pruned and optimized using built-in 10-fold cross-validation to minimize the relative misclassification of cases while protecting against overfitting. C-indices, a confusion matrix, and a receiver operating characteristic curve were generated to evaluate performance of the final decision tree.

## Results

### Cohort Assembly and Baseline Characteristics

**Figure 1:**
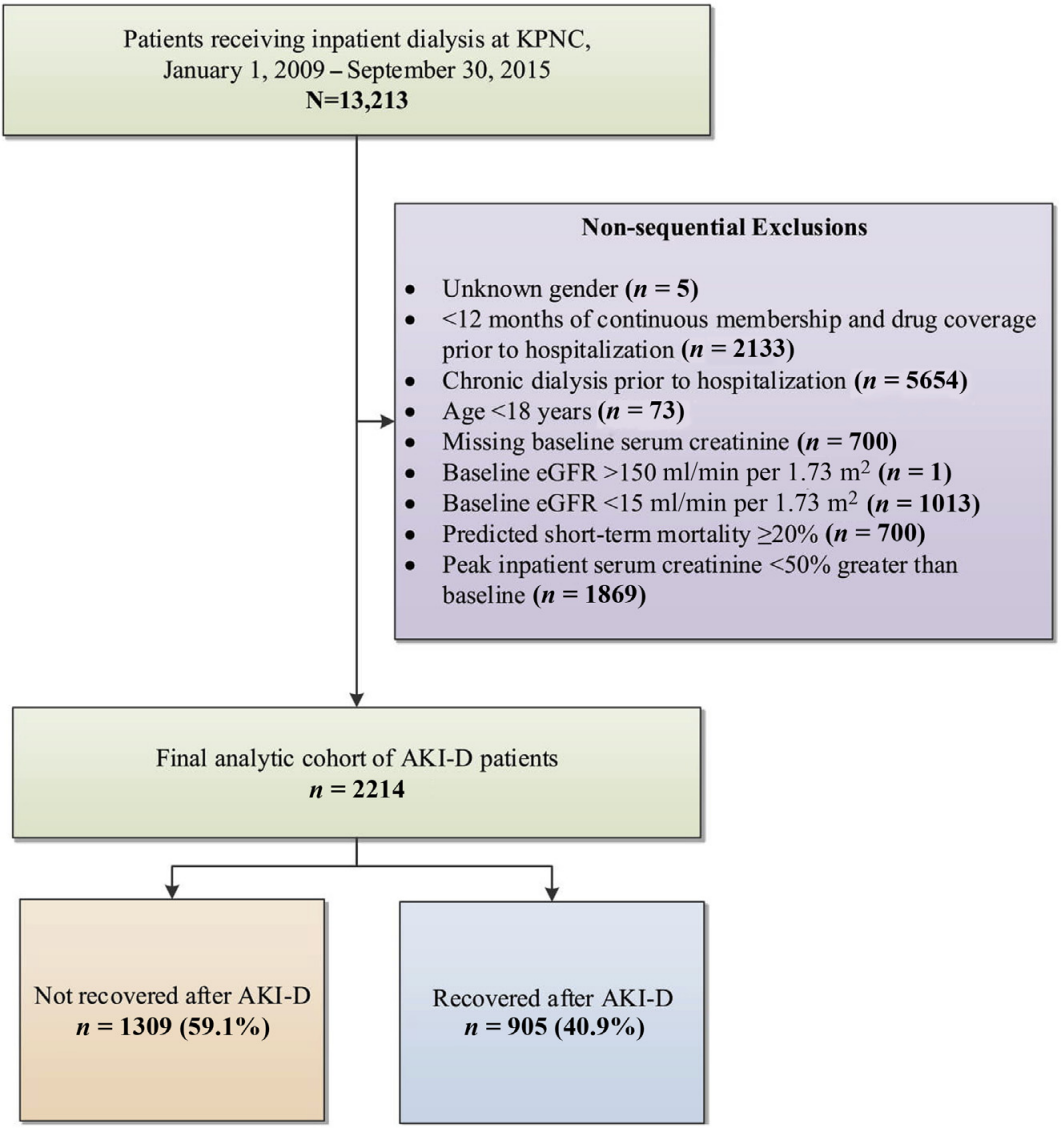
Cohort assembly for adults experiencing dialysis-requiring acute kidney injury (AKI-D). eGFR, estimated glomerular filtration rate; KPNC, Kaiser Permanente Northern California.

We initially identified 13,213 adult patients who received inpatient RRT. After excluding patients who received chronic dialysis before hospitalization, were of age <18 years, had unknown gender, had <12 consecutive months of membership or drug coverage before the index hospitalization, had no baseline serum creatinine concentration, had baseline eGFR >150 or <15 ml/min per 1.73 m^2^, had a predicted probability of inpatient mortality ≥20%, or had <50% increase in peak inpatient serum creatinine concentration compared with preadmission baseline, we had a final analytic cohort of 2214 patients with AKI-D (Figure 1).

Mean age was 67.1 years, 40.8% were women, and 54.0% were white. Overall, 905 (40.9%) patients recovered within 90 days of RRT initiation. Of the patients who did not recover, 731 (55.8%) died while still dialysis-dependent. Selected candidate predictors are presented in Table 1; the remaining additional candidate predictors are reported in Supplementary Table S1. Compared with patients who did not recover, patients who recovered were younger and less likely to have a history of heart failure or chronic liver disease. Those who recovered had a higher body mass index, higher baseline eGFR, less proteinuria, and higher preadmission hemoglobin level (Table 1).

### Logistic Regression

**Figure 2:**
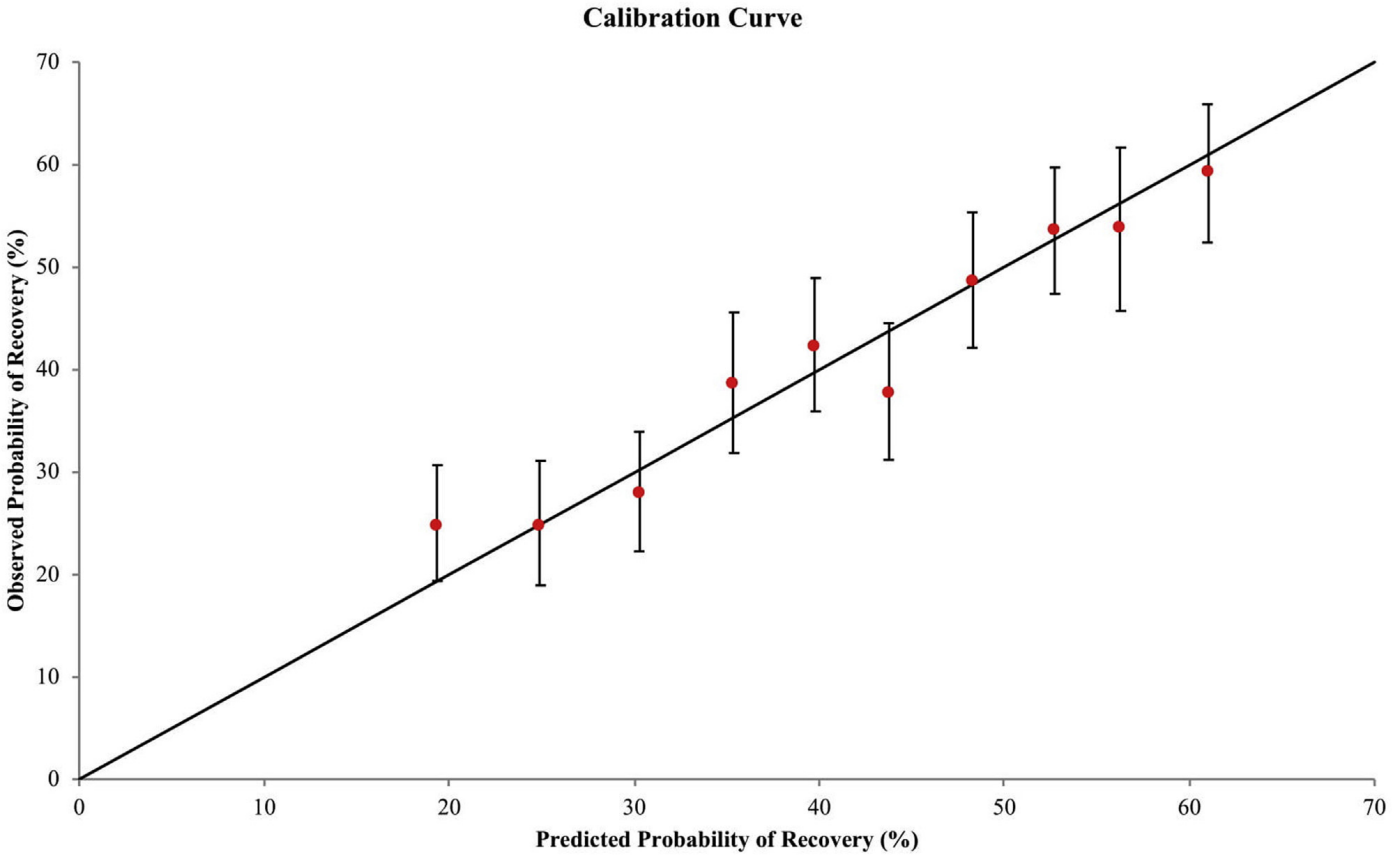
Calibration curve for predicted and observed probabilities of renal recovery using 10-fold cross-validation, by decile of predicted probability.

In 1000 bootstrap samples of the analytic cohort, 4 predictors were chosen by stepwise regression in >75% of samples: baseline (preadmission) eGFR (all 1000 samples), preadmission hemoglobin level (954 samples), history of chronic liver disease (863 samples), and age (802 samples). The c-index of a model with these predictors, obtained using observed and predicted values from 10-fold cross-validation, was 0.64. The correlation coefficient (*R*) between observed and predicted probabilities of recovery, plotted by decile of predicted probability of recovery, was high at 0.97 (Figure 2). Predicted recovery probabilities ranged from 9% to 22% in the lowest decile to 58% to 66% in the highest decile. Using the full analytic cohort, we obtained odds ratios for recovery for the 4 chosen predictors using logistic regression (Table 2).

In our sensitivity analysis that did not exclude patients with predicted probability of inpatient mortality ≥20%, the same predictors were chosen using our bootstrapping and cross-validation approach, with no significant change in the c-index (0.645).

In an additional sensitivity analysis, results did not materially differ if serum creatinine concentration was used instead of eGFR (c-index 0.646), and the same predictors were selected.

### CART Analysis

**Figure 3:**
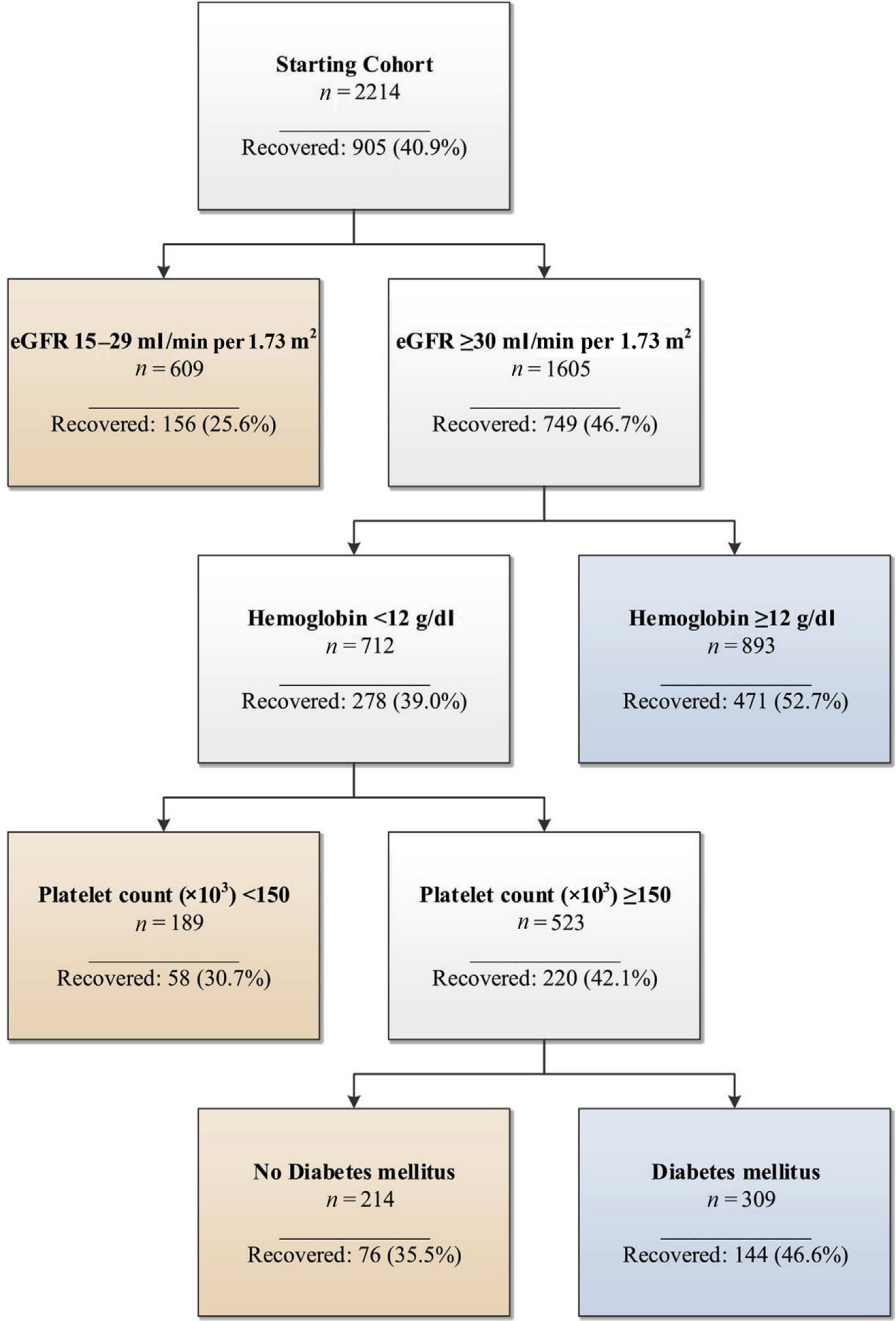
Classification and regression tree decision tree for recovery after dialysis-requiring acute kidney injury. eGFR, estimated glomerular filtration rate.

The final decision tree included 4 nodes: eGFR ≥30 ml/min per 1.73 m^2^, preadmission hemoglobin <12.0 g/l, preadmission platelet count ≥150,000/μl, and history of diabetes mellitus (Figure 3). CART subdivided the cohort into 5 risk groups with recovery probabilities ranging from 25.6% to 52.7%. The c-index obtained from 10-fold cross-validation was 0.61.

## Conclusion

We developed and cross-validated a parsimonious logistic regression model for recovery after AKI-D using variables that are routinely available in clinical practice. To our knowledge, our study is the first to develop a recovery prediction model that uses clinical data from a diverse, community-based cohort. Although novel, simple to use, and having excellent calibration (i.e., ability to predict absolute risk accurately), our model demonstrated only modest discrimination, which limits its clinical utility.

We believe that our model’s disappointing discrimination highlights how very challenging it is to distinguish relatively between patients with higher versus lower chances of recovery in a real-world clinical setting using information currently available to physicians. This finding is entirely concordant with our recent report that approximately 1 in every 24 patients registered as having ESRD in the U.S. Renal Data System recovered to discontinue dialysis and likely had AKI-D misclassified as permanent kidney failure instead.^43^ This dilemma has population-level implications, particularly in the United States where reimbursement policies for dialysis services have historically differed depending on whether a patient is designated as having AKI-D or ESRD. The Centers for Medicare and Medicaid Services only reinstated reimbursement for dialysis provided to AKI-D outpatients in 2017, and payments for skilled nursing facility residents with AKI-D only started in 2018.^44^ Furthermore, there has been considerable debate regarding whether patients with AKI-D should be included in the ESRD Quality Incentive Program, which affects payments to ESRD facilities.^45^ Because optimal clinical management of patients with AKI-D and of patients with ESRD differ, the American Society of Nephrology^46^ and Renal Physicians Association^47^ have strongly advocated against including patients with AKI-D in the ESRD Quality Incentive Program. Our results argue that policies based on assuming that physicians are able to predict accurately whether a patient truly has ESRD or not at the time of RRT initiation may be unwise and unrealistic. A better approach may be to recognize this diagnostic uncertainty and modify policy accordingly, such as by asking physicians to certify patients as having ESRD or not only after a certain period has elapsed.

Our findings that younger age and higher baseline eGFR are strong predictors for recovery are consistent with prior studies.^12^, ^13^, ^14^, ^15^ The fact that chronic liver disease is associated with reduced chances for recovery may be due to compromised renal perfusion in the setting of hepatorenal physiology.^48^ We speculate that our finding that higher preadmission hemoglobin level predicts recovery may reflect higher preadmission eGFR (because hemoglobin level and eGFR may be correlated, and our hemoglobin categories were narrower than our eGFR categories) or generally better health status overall.

Strengths of our study include the large, contemporary cohort of patients with AKI-D with broad demographic diversity. Although most other studies have included only patients in intensive care units,^5^, ^12^, ^16^, ^49^, ^50^ our cohort included both intensive care unit and medical ward patients in 21 medical centers across Northern California.^23^ KPNC’s integrated health care delivery system offers the advantage of being able to track recovery longitudinally both during the AKI-D hospitalization and in the outpatient setting after hospital discharge. In addition, it provides the unique opportunity to ascertain preadmission comorbidities, medication use, and laboratory values. In contrast, many AKI epidemiology studies^2^, ^51^, ^52^ have had limited ascertainment of key clinical covariates before hospitalization. We used rigorous criteria to identify AKI-D cases and were careful about requiring patient survival for ≥4 weeks after dialysis discontinuation to avoid misclassification of withdrawal of care as recovery. Our approach of anchoring recovery from time of dialysis initiation rather than time of hospital discharge also enhances our results’ generalizability compared with prior studies because timing of hospital discharge may be affected by social and systems-based factors unrelated to the natural history of AKI-D. Finally, we used 2 modeling techniques to try to enhance our ability to predict renal recovery.

Several limitations should be noted. Because predicting recovery is clinically relevant only once patients are improving, typically in the denouement phase of acute illness, the ideal study population in which to derive a prediction rule should include only patients with AKI-D who reach this juncture in their hospitalizations. However, it is not possible to identify this stage of disease trajectory in large database studies such as ours. We therefore limited our study population to the subset of patients with AKI-D who could be readily identified clinically as not having excessively high predicted inpatient mortality risk. We did not want to limit our study population only to AKI-D survivors because such an approach would use retrospective conditioning. We required that all cases of AKI-D have a ≥50% increase in serum creatinine over the preadmission baseline value to reduce misclassification of progressive CKD as AKI. However, as a result, we may have excluded some cases of true AKI-D (e.g., a cardiac surgery patient who is anuric and volume-overloaded postoperatively may initiate RRT in the setting of true AKI but may not meet this threshold to be included in our analysis). We defined recovery as a dichotomous outcome based on RRT dependence and did not estimate magnitude of recovery (e.g., full vs. partial recovery). Although functional recovery beyond RRT dependence is certainly important, definitions for recovery are variable,^22^ and serum creatinine levels may be affected by dilution from fluid accumulation^53^ and fluctuating creatinine production,^54^, ^55^ which makes evaluating recovery as a change in serum creatinine concentration from before to after the acute illness less straightforward. Furthermore, in current practice, most physicians do not systematically ascertain for recovery. Another limitation of our study was that not all clinical details related to the AKI-D hospitalizations were available. We were missing information regarding etiology of preexisting CKD, indication for RRT initiation, initial RRT modality (e.g., intermittent vs. continuous therapy), physiologic variables at time of RRT initiation including APACHE score and urine output, inpatient medication use, and setting of AKI (e.g., sepsis or postsurgery); however, there is no definitive evidence that dialysis duration,^12^, ^56^ dialysis dose,^57^, ^58^ choice of dialysis membrane,^59^ RRT modality,^9^, ^12^, ^56^, ^59^, ^60^, ^61^, ^62^, ^63^, ^64^ timing of dialysis initiation,^65^, ^66^, ^67^, ^68^ or medications such as diuretics^69^, ^70^, ^71^ are associated with chances of recovery. Although the specific etiology of AKI-D was also unavailable in our dataset, prior chart review of KPNC medical records by a board-certified nephrologist of similar cases showed that almost all were due to acute tubular necrosis.^13^, ^15^ We were not able to examine whether the Charlson comorbidity index and APACHE II score were predictive for recovery, as previously reported,^18^ because not all of the components were available. However, the vast majority of these scores’ parameters are accounted for in the validated KPNC inpatient mortality score that was used instead.^26^ Finally, while we used a 10-fold cross-validation approach, our results should be further validated in other external patient populations that have similarly broad diversity in sociodemographic features and comorbidity burden.

In conclusion, we have developed and cross-validated a prediction model for recovery after AKI-D, but our findings reiterate the need for better clinical prediction tools. Although our model demonstrates excellent calibration, its modest discrimination may reflect the complexity of factors affecting recovery. The addition of selected biomarkers to clinical parameters may prove useful in enhancing predictive models in the future.^18^, ^72^, ^73^ Future research is needed to enhance our predictive abilities as well as to examine potential treatments that may enhance or expedite recovery after AKI-D.

## Disclosure

All the authors declared no competing interests.

## Supplementary Material

Table S1 Additional baseline characteristics of study cohort, stratified by renal recovery status.

Appendix S1 List of hospitals in which the study population was treated.
