# Supplementary material for: Predicting Renal Recovery After Dialysis-Requiring Acute Kidney Injury
Source: Kidney Int Rep. 2019 Jan 28;4(4):571–81. doi: 10.1016/j.ekir.2019.01.015 (PMC6451155; doi:10.1016/j.ekir.2019.01.015)
Supplement: Table S1 — Additional baseline characteristics of study cohort, stratified by renal recovery status. [file mmc1.docx]

**Supplementary Table 1.** Additional baseline characteristics of adults with dialysis-requiring acute kidney injury, stratified by renal recovery status.

|  | **Overall** | **Not recovered** | **Recovered** |  |
| --- | --- | --- | --- | --- |
| **Variable**^a^ | **(N=2,214)** | **(N=1,309)** | **(N=905)** | **P-value** |
| **Smoking status**, n (%) |  |  |  | 0.68 |
| Current Smoker | 164 (7.4) | 92 (7.0) | 72 (8.0) |  |
| Former smoker | 1027 (46.4) | 613 (46.8) | 414 (45.7) |  |
| Nonsmoker | 1023 (46.2) | 604 (46.1) | 419 (46.3) |  |
| **Medical history**, n (%) |  |  |  |  |
| Percutaneous coronary intervention | 113 (5.1) | 72 (5.5) | 41 (4.5) | 0.31 |
| Atrial flutter or fibrillation | 464 (21.0) | 292 (22.3) | 172 (19.0) | 0.06 |
| Mitral or aortic valvular disease | 334 (15.1) | 204 (15.6) | 130 (14.4) | 0.43 |
| Peripheral arterial disease | 67 (3.0) | 39 (3.0) | 28 (3.1) | 0.88 |
| Dyslipidemia | 1737 (78.5) | 1033 (78.9) | 704 (77.8) | 0.53 |
| Venous thromboembolism | 60 (2.7) | 34 (2.6) | 26 (2.9) | 0.69 |
| Hyperthyroidism | 21 (0.9) | 7 (0.5) | 14 (1.5) | **0.02** |
| Hypothyroidism | 381 (17.2) | 236 (18.0) | 145 (16.0) | 0.22 |
| Chronic lung disease | 751 (33.9) | 453 (34.6) | 298 (32.9) | 0.41 |
| Dementia | 40 (1.8) | 29 (2.2) | 11 (1.2) | 0.08 |
| Depression | 437 (19.7) | 251 (19.2) | 186 (20.6) | 0.42 |
| Chronic lung disease | 751 (33.9) | 453 (34.6) | 298 (32.9) | 0.41 |
| Hospitalized bleed | 156 (7.0) | 114 (8.7) | 42 (4.6) | **<0.001** |
| **Systolic blood pressure** [mm Hg] | 127.4 (21.0) | 127.6 (21.8) | 127.1 (19.8) | 0.64 |
| **Pre-admission medication use**, n (%) |  |  |  |  |
| Alpha blocker | 375 (16.9) | 227 (17.3) | 148 (16.4) | 0.54 |
| Beta blocker | 1223 (55.2) | 729 (55.7) | 494 (54.6) | 0.61 |
| Calcium channel blocker | 697 (31.5) | 410 (31.3) | 287 (31.7) | 0.85 |
| Aldosterone receptor antagonist | 206 (9.3) | 143 (10.9) | 63 (7.0) | **0.002** |
| Nitrates | 330 (14.9) | 231 (17.6) | 99 (10.9) | **<0.001** |
| Vasodilators | 397 (17.9) | 288 (22.0) | 109 (12.0) | **<0.001** |
| Anti-arrhythmic drug | 91 (4.1) | 53 (4.0) | 38 (4.2) | 0.86 |
| Statin | 1330 (60.1) | 775 (59.2) | 555 (61.3) | 0.32 |
| Other lipid-lowering agent | 142 (6.4) | 76 (5.8) | 66 (7.3) | 0.16 |
| Non-aspirin antiplatelet agent | 190 (8.6) | 111 (8.5) | 79 (8.7) | 0.84 |
| **Laboratory values** |  |  |  |  |
| Pre-admission total cholesterol [mg/dL] |  |  |  |  |
| >240 | 117 (5.3) | 66 (5.0) | 51 (5.6) | 0.10 |
| 200-240 | 210 (9.5) | 110 (8.4) | 100 (11.0) |  |
| <200 | 1336 (60.3) | 791 (60.4) | 545 (60.2) |  |
| Pre-admission high-density lipoprotein [mg/dL] |  |  |  |  |
| >=60 | 228 (10.3) | 136 (10.4) | 92 (10.2) | 0.50 |
| 50-59 | 232 (10.5) | 127 (9.7) | 105 (11.6) |  |
| 40-49 | 436 (19.7) | 253 (19.3) | 183 (20.2) |  |
| 35-39 | 306 (13.8) | 186 (14.2) | 120 (13.3) |  |
| <35 | 428 (19.3) | 247 (18.9) | 181 (20.0) |  |
| Pre-admission low-density lipoprotein [mg/dL] |  |  |  |  |
| >=200 | 39 (1.8) | 26 (2.0) | 13 (1.4) | **0.04** |
| 160-199 | 65 (2.9) | 31 (2.4) | 34 (3.8) |  |
| 130-159 | 138 (6.2) | 69 (5.3) | 69 (7.6) |  |
| 100-129 | 299 (13.5) | 175 (13.4) | 124 (13.7) |  |
| 70-99 | 646 (29.2) | 374 (28.6) | 272 (30.1) |  |
| <70 | 618 (27.9) | 384 (29.3) | 234 (25.9) |  |
| Inpatient hemoglobin [g/dL] | 9.7 (1.7) | 9.6 (1.7) | 9.7 (1.8) | 0.12 |
| Inpatient serum albumin [g/dL] | 2.7 (0.7) | 2.6 (0.7) | 2.7 (0.7) | 0.09 |
| Inpatient platelet count [x10^3^/μL] | 184.8 (112.5) | 181.0 (108.4) | 190.3 (118.2) | 0.06 |

^a^Mean (SD) unless otherwise indicated.
