# Supplementary material for: Predicting Renal Recovery After Dialysis-Requiring Acute Kidney Injury
Source: Kidney Int Rep. 2019 Jan 28;4(4):571–81. doi: 10.1016/j.ekir.2019.01.015 (PMC6451155; doi:10.1016/j.ekir.2019.01.015)
Supplement: Appendix S1 — List of hospitals in which the study population was treated. [file mmc2.docx]

**Appendix.** List of hospitals in which the study population was treated.

Antioch Medical Center

Vacaville Medical Center

Vallejo Medical Center

Walnut Creek Medical Center

Fremont Medical Center

Oakland Medical Center

Richmond Medical Center

San Leandro Medical Center

Fresno Medical Center

Manteca Medical Center

Modesto Medical Center

Roseville Medical Center

Sacramento Medical Center

South Sacramento Medical Center

Santa Clara Homestead Medical Center

Santa Teresa/San Jose Medical Center

San Francisco Medical Center

San Rafael Medical Center

Santa Rosa Medical Center

South San Francisco Medical Center

Redwood City Medical Center
